# Effects of Aging Treatment on the Microstructure and Mechanical Properties of Rene 41 Alloy and the Strengthening Mechanisms

**DOI:** 10.3390/ma18071579

**Published:** 2025-03-31

**Authors:** Xianguang Zhang, Haoran Han, Pingmei Tang, Yang Zhou, Dongping Xiao, Jianhui Fu, Jiajun Chen, Shouli Feng, Jian Zhang

**Affiliations:** 1 School of Metallurgical and Ecological Engineering, University of Science and Technology Beijing, Beijing 100083, China; 2 Chengdu Advanced Metal Materials Industrial Technology Institute Co., Ltd., Chengdu 610300, China

**Keywords:** Rene 41 alloy, aging treatment, microstructure, strengthening mechanisms, precipitation strengthening

## Abstract

Aging treatment plays a significant role in altering the mechanical properties of superalloys; however, the influences of aging on the mechanical performance of Rene 41 alloy and its strengthening mechanisms remain unclear. In this study, the effects of aging on the microstructure and mechanical properties of forged Rene 41 alloy were systematically investigated through experimental and theoretical analyses. It was found that aging conditions have significant influences on the grain structure, γ′ characteristics, and tensile properties of the alloy. The size of secondary γ′ increased from 20 nm to 69 nm with elevated aging temperature and prolonged aging time, while their volume fraction initially increased and then decreased. Notably, the γ′ maintained a coherent interface with the matrix even after high-temperature aging at 860 °C. An optimal strength–ductility balance was achieved by aging at 760 °C for 16 h. An experimentally verified strengthening model was used for understanding the strengthening mechanisms of the alloy aging at varying conditions. Precipitation strengthening was identified as the dominant strengthening mechanism, substantially contributing to the overall yield strength. The precipitation strengthening mainly belongs to the strong pair-coupling cutting mechanism rather than the Orowan bypass mechanism. This study concludes that a rational aging treatment regime can significantly optimize the comprehensive properties of Rene 41 alloy, providing theoretical support for its application in aerospace component manufacturing.

## 1. Introduction

As the power source of the aircraft, aviation engines play a critical role in the aerospace field [1]. The working environment of aeroengines is high in temperature and pressure, which imposes stringent requirements on the component’s performance [2]. Rene 41, a hard-to-deform superalloy with excellent high-temperature strength and oxidation resistance, is a key material widely used in engine components such as turbine blades, turbine disks, and combustion chambers [3,4,5]. With the increasing thrust-to-weight ratio of aeroengines, the turbine inlet temperature continues to rise, and higher requirements are put forward for materials performance [6,7]. The alloy performance can be effectively enhanced through heat treatment by modifying its microstructures [8,9]. For superalloys, heat treatment primarily involves two steps: solution treatment and aging treatment. Solution treatment aims to dissolve the coarse precipitates, while the aging treatment re-precipitates the precipitates and improves the structural stability of alloy under the service conditions. For example, the size, shape, and distribution of the strengthening phases can be optimized, the stability of which can be improved by aging [10]. Therefore, investigating the effects of aging treatment on Rene 41 alloy is essential for optimizing its elevated-temperature mechanical properties.

During aging treatment, superalloys undergo significant microstructural changes, including the coarsening and morphology change of γ′, grain growth, and transformation of precipitates types. For example, Guo [11] observed that the γ′ size is increased from 9 nm to 51 nm during aging at 700 °C in an Fe-based superalloy. Zhang [12] reported that the GH105 alloy hardness initially decreased and then increased with rising aging temperature, due to the grain growth and dissolution of grain boundary carbides. Li [13] studied two nickel-based single-crystal superalloys with varying γ′ contents and found that, while the γ′ size increased with aging time, its morphology remained stable; however, alloys with higher γ′ content formed TCP phases during aging. It was also reported that the γ′ morphology was changed from spherical to cubic and its volume fraction was reduced by the prolonged aging in Ni–Fe–Cr-based superalloys, leading to strength degradation [14]. Liu [15] observed γ′ coarsening, η phase formation, and *M*C carbide transformation during the long-term aging. Notably, aging induced microstructural changes vary significantly across alloys, sometimes yielding contradictory conclusions. The existing studies on aging treatment can only provide references for the Rene 41 alloy, and systematic research on its microstructure evolution and mechanical properties remain imperative.

Furthermore, establishing a strengthening model is crucial for correlating microstructure with mechanical properties and elucidating the strengthening mechanisms under different aging conditions. For instance, Zhang [16] developed a quantitative model for selective laser-melted Inconel 718 alloy, integrating the dislocation density, precipitates, and microstructural anisotropy to highlight the dominance of coherency strengthening. Wen [17] proposed a comprehensive model for FGH4113A alloy, quantifying contributions from grain boundary, solid solution, and precipitation strengthening with the latter accounting for over 60% of yield strength. In iron-based superalloys, Zhang [18] utilized the Bailey–Hirsch model to assess the dislocation hardening and Orowan mechanisms, while estimating nanotwin strengthening by subtracting known contributions. An increase in twin boundary has been shown to increase the strength of alloys, which is comparable to that of grain refinement in superalloy [19] and enhancing the strain-hardening effect in steel [20]. As for the γ’ size influences on the precipitation strengthening mechanism, there is a debate on the influences of γ’ sizes on the cutting or Orowan bypass mechanisms [21,22] probably due to different alloy systems and alloys state. Besides the above efforts, the research on the strengthening mechanisms for the aged Rene 41 alloy remains scarce. Therefore, this work aimed to study the mechanical responses of Rene 41 alloy under varying aging conditions and clarifying the underlying strengthening mechanisms by using the experimentally verified strengthening model.

## 2. Experimental Procedures

**Figure 1:**
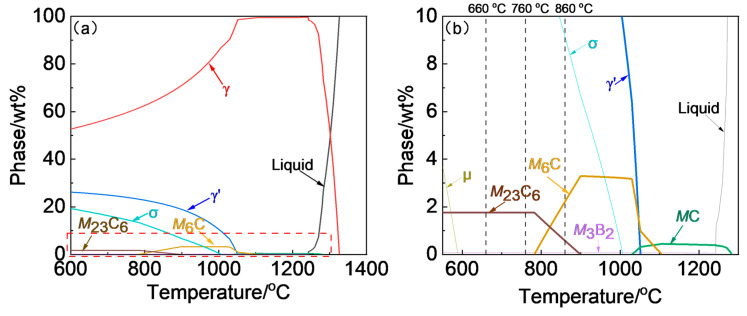
(**a**) Equilibrium phase diagram and (**b**) magnified view of the dashed-line region in (**a**) for Rene 41 alloy [23].

**Figure 2:**
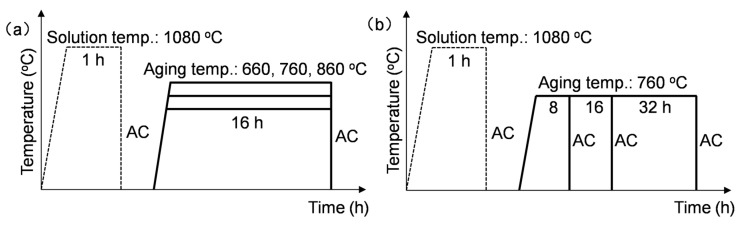
Thermal histories for the heat treatments under varying (**a**) aging temperature and (**b**) aging periods for the forged Rene 41 superalloy.

The Rene 41 superalloy used in this study was forged after homogenization and clogging treatments. The chemical composition of the as-forged alloy is presented in Table 1. Additionally, the equilibrium phase diagram, which was calculated using JMatPro (version 9.0, Sente Software, University of Surrey, The United Kingdom of Great Britain and Northern Ireland), is depicted in Figure 1. As mentioned in the Introduction part, the aim of aging treatment is to re-precipitate the precipitates and improve the structural stability of the superalloy under service conditions. For Rene 41 alloy, the service temperature generally ranges from 650 °C to 850 °C, and the typical service temperature is around 760 °C. Consequently, 660 °C and 860 °C were selected, which are 100 °C below or above the typical service temperature. The experimentally designed aging temperatures are displayed in the phase diagram (Figure 1b) for comparisons. The detailed thermal history for studying the influences of aging temperature and time is shown in Figure 2. It needs to be pointed out that the aging period, ranging from 8 h to 32 h, is primarily determined by the previse study of the present authors [23]. The solution treatment conditions were the same for all cases, which were determined by the previous research [23].

The heat treatments were carried out in a high-temperature box-type resistance furnace. The samples cut from the heat-treated samples with the size of 10 mm × 5 mm × 2 mm were used for the microstructure characterization. Chemical etching (3 g CuSO_4_ + 40 mL HCl + 3 mL H_2_SO_4_, 30–40 s, Sinopharm Chemical Reagent Co., Ltd., Shanghai, China) was preformed after mechanical polishing and grinding to reveal the grain boundaries and γ′. Optical microscope (OM, CX40M, SUNNY GROUP, Yuyao City, China) and scanning electron microscope (SEM, JSM 7200F, JEOL, Tokyo, Japan) equipped with an X-ray energy spectrometer (EDS, NS7, JEOL, Tokyo, Japan) were used for the microstructure characterization. Some samples were characterized by electron backscattered diffraction (EBSD, MIRA3 LMH, TESCAN, Shanghai, China) and transmission electron microscopy (TEM, Thermofisher Talos F200X, Thermo Fisher Scientific, Waltham, MA, USA) equipped with EDS. To remove the surface damage after mechanical polishing, electrolytic polishing (20 mL HCl + 80 mL C_2_H_5_OH, 20 V, 10 s) was conducted prior to EBSD analyses. Thin-film TEM samples were fabricated by twin-jet electropolishing using a Struers Tenupol-5 machine with an electrolyte solution of 10% perchloric acid (HClO_4_) and 90% ethanol (C_2_H_5_OH) at 20 V and −25 °C. The volume fraction and size of the precipitated phase were analyzed using the ImageJ software (version 1.53e, National Institutes of Health, Bethesda, MD, USA). The high-temperature tensile tests were conducted using a universal testing machine, in accordance with the standard ASTM E21-20 [24]. The high-temperature tensile tests were performed at 760 °C at a rate of 1 mm/min, with a holding time of 5 min before the tensile test. For re-productivity, tensile tests were repeated three times, and the errors were obtained based on the results of the three repeats to eliminate the interference of randomness. The 0.2% offset method (*σ*_0.2_) was applied in this work for determining the yield strength.

## 3. Experimental Results and Discussion

### 3.1. Initial Structure Characterization

**Figure 3:**
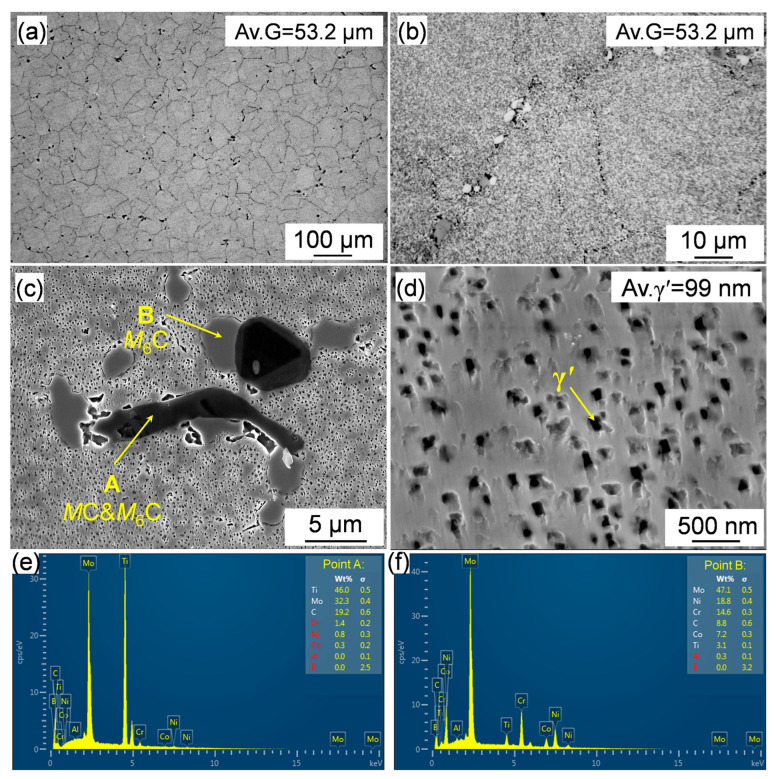
(**a**,**b**) OM and (**c**,**d**) SEM images displaying the carbides and γ′ in the as-forged Rene 41 alloy; (**e**,**f**) EDS point analyses of the regions labeled A and B in image (**c**).

The microstructure of the Rene 41 alloy in its as-forged state prior to heat treatment, is illustrated in Figure 3, along with the quantified average grain size. As shown in Figure 3a, the grain structure is relatively uniform and the average grain size is around 53.2 μm. A large number of blocky chain-like precipitates was observed near the grain boundaries (Figure 3b). SEM images and the EDS point analyses results are displayed in Figure 3c,e,f. The black precipitates in the SEM image are rich in Mo and Ti, while the gray ones are rich in Mo. According to previous research [25], the black and gray precipitated phases are *M*C + *M*_6_C and *M*_6_C carbides, respectively. Furthermore, coarse γ′ precipitates are clearly observed (Figure 3d), and the size was measured to be approximately 99 nm. The presence of coarse carbides and γ′ may deteriorate the mechanical properties [26]. Therefore, it is necessary to dissolve the coarse γ′ and carbides by solution treatment and re-precipitate fine ones by aging to enhance the mechanical properties.

**Figure 4:**
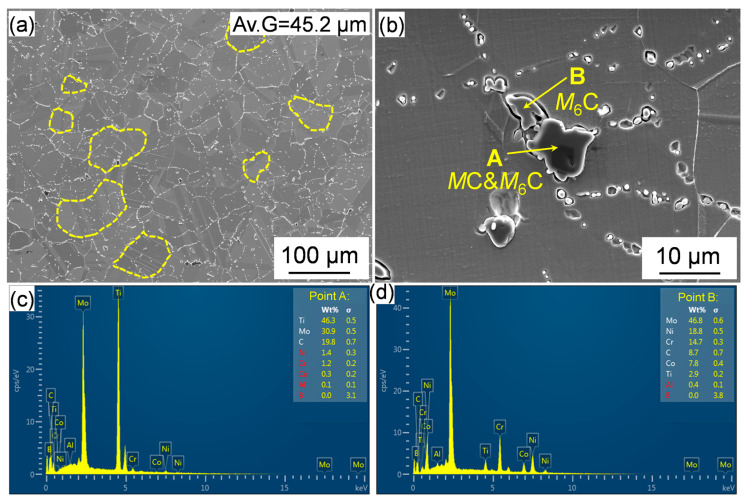
SEM images showing the grains (**a**) and carbides (**b**) after the solution treatment; (**c**,**d**) EDS analyzed results of the areas A and B in SEM image shown in (**b**).

The SEM images of the alloy after solution treatment at 1080 °C for 1 h and air-cooling is displayed in Figure 4. The average grain size of the solution-treated Rene 41 alloy is approximately 45.2 μm. The coarse grain boundary carbides have been largely dissolved and some residual coarse carbides were occasionally observed. According to the EDS analysis, the black and gray carbides are *M*C + *M*_6_C and *M*_6_C, respectively. In addition, the coarse γ′ in the as-forged alloy were vanished. The γ′ is predicted to be completely dissolved at approximately 1051.7 °C, according to the equilibrium phase diagram shown in Figure 1. Therefore, solution treatment at 1080 °C, which is above the solvus temperature, can ensure the complete dissolution of γ’ thermodynamically. Indeed, the same result was obtained in the previous work [23] that the coarse primary γ′ in the initial structure has been completely dissolved by using the same solution treatment condition with the current work.

### 3.2. Influences of Aging Temperature

**Figure 5:**
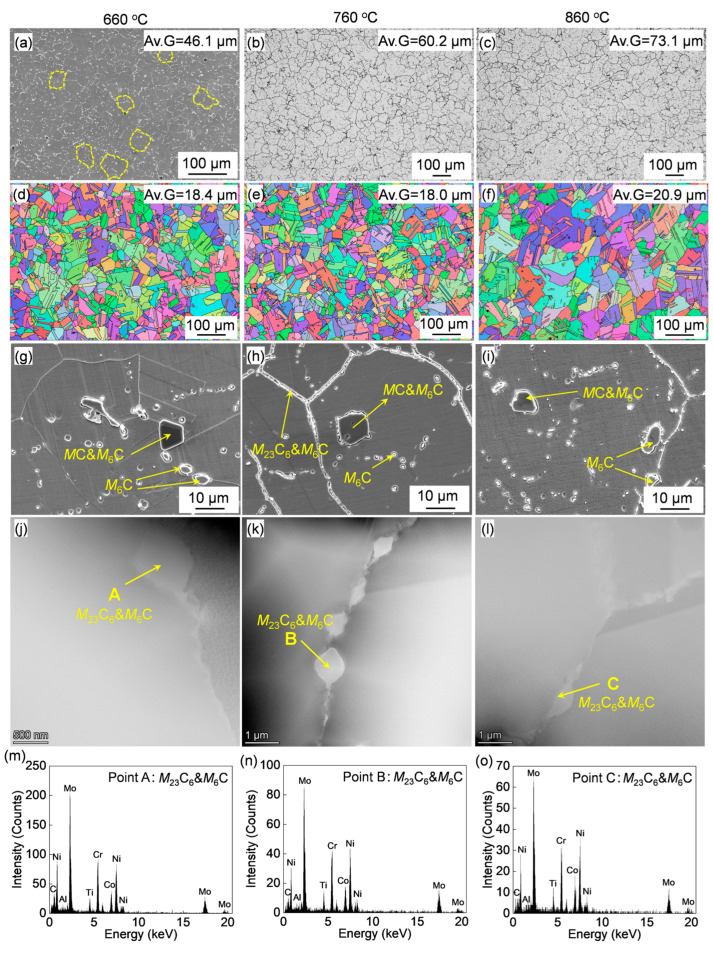
(**a**,**g**–**i**) SEM images, (**b**,**c**) OM images, and (**d**–**f**) IPF maps of the forged Rene 41 alloy after aging at different temperatures; (**j**–**l**) HAADF images and (**m**–**o**) the corresponding EDS analyzed results.

The OM, SEM, and TEM images of the solution-treated alloy after aging at varying temperatures are shown in Figure 5. The grain structure after aging at 660 °C could not be easily observed by OM; therefore, SEM observation was performed instead (Figure 5a). It can be observed that the average grain size (excluding twins) for the alloy aged at 660 °C is 46.1 μm, which is close to that of the solution-treated state. This indicates that aging at 660 °C has a negligible influence on grain size. The average grain sizes of the alloy aged at 760 °C and 860 °C were measured to be 60.2 μm and 73.1 μm, respectively. The grains gradually coarsened with increasing aging temperature. In addition, local grain coarsening occurred during high-temperature aging, leading to a deterioration in grain structure uniformity. These results demonstrate that the microstructure of the alloy is significantly influenced by the aging temperature.

In addition to grain boundaries, a large number of twins were observed as well (Figure 5a–c). As mentioned in the Introduction part, twin boundaries have significant influences on the service life and strengthening of alloys [19,27]. The typical IPF image analyzed by EBSD and the average grain size with consideration of twin boundaries were quantified using the equivalent circular diameter method with the help of AztecCrystal (v.2.1) software are shown in Figure 5d–f. The average grain size was significantly refined by the formation of twins.

The carbides are rarely distributed on the grain boundaries after aging at 660 °C (Figure 5d–f), which are primarily distributed intragranularly. In contrast, the fine grain-boundary carbides are well developed after aging at 760 °C, which exhibited a thicker ‘grain boundary’ under SEM observation (Figure 5e). As the aging temperature further increases to 860 °C, the number of grain-boundary carbides was decreased. Therefore, aging at 760 °C would yield excellent grain boundary strengthening among the three aging conditions. The HAADF and STEM-EDS analyses are shown in Figure 5g–l. The gray precipitates (labeled A, B, C) observed under HAADF are enriched in Mo and Cr elements, which are identified as *M*_23_C_6_ + *M*_6_C carbides according to previous studies [25]. The formation of grain-boundary *M*_23_C_6_ carbide is due to the grain boundary segregation of elements during the cooling process after solution treatment and subsequent aging process [28].

**Figure 6:**
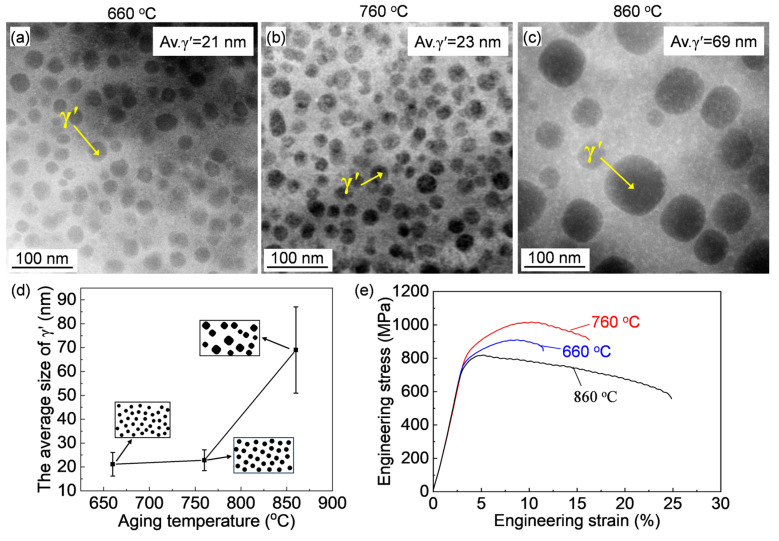
(**a**–**c**) HAADF images of the alloy after aging treatments at different temperatures, and (**d**) schematic illustrations of the γ’ morphology and size, as well as the quantitative analysis of changes in γ’ size with varying aging-treatment temperatures. (**e**) Stress–strain curves of the alloy at 760 °C after aging treatments at different temperatures.

The characteristics of γ′ after aging at varying temperatures were characterized by TEM, and typical HAADF images are shown in Figure 6. According to the above results, the coarse primary γ′ in the as-forged alloy was completely dissolved by solution treatment. Therefore, the fine γ′ observed after aging consists of re-precipitated secondary γ′. The quantitatively analyzed changes in γ′ diameter, along with a schematic illustration of the variations in size and morphology with aging temperature, are presented in Figure 6d. The γ′ size gradually increased from 21 nm to 69 nm, and the morphology tended to change from spherical to quasi-square as the aging temperature increased. It is evident that γ′ was significantly coarsened by high-temperature aging at 860 °C.

The mechanical properties at the elevated-temperature of 760 °C for the alloys aged at varying temperatures are displayed in Figure 6e and the specific mechanical properties were summarized in Table 2. The aged alloy exhibited varying mechanical responses. The yield strength increases at first and then decreases as the aging temperature was increased from 660 °C to 860 °C. While the elongation keeps increasing with the increase in aging temperature. These findings indicate that aging treatment has a significant influence on the mechanical properties of the alloy.

### 3.3. Influences of Aging Time

**Figure 7:**
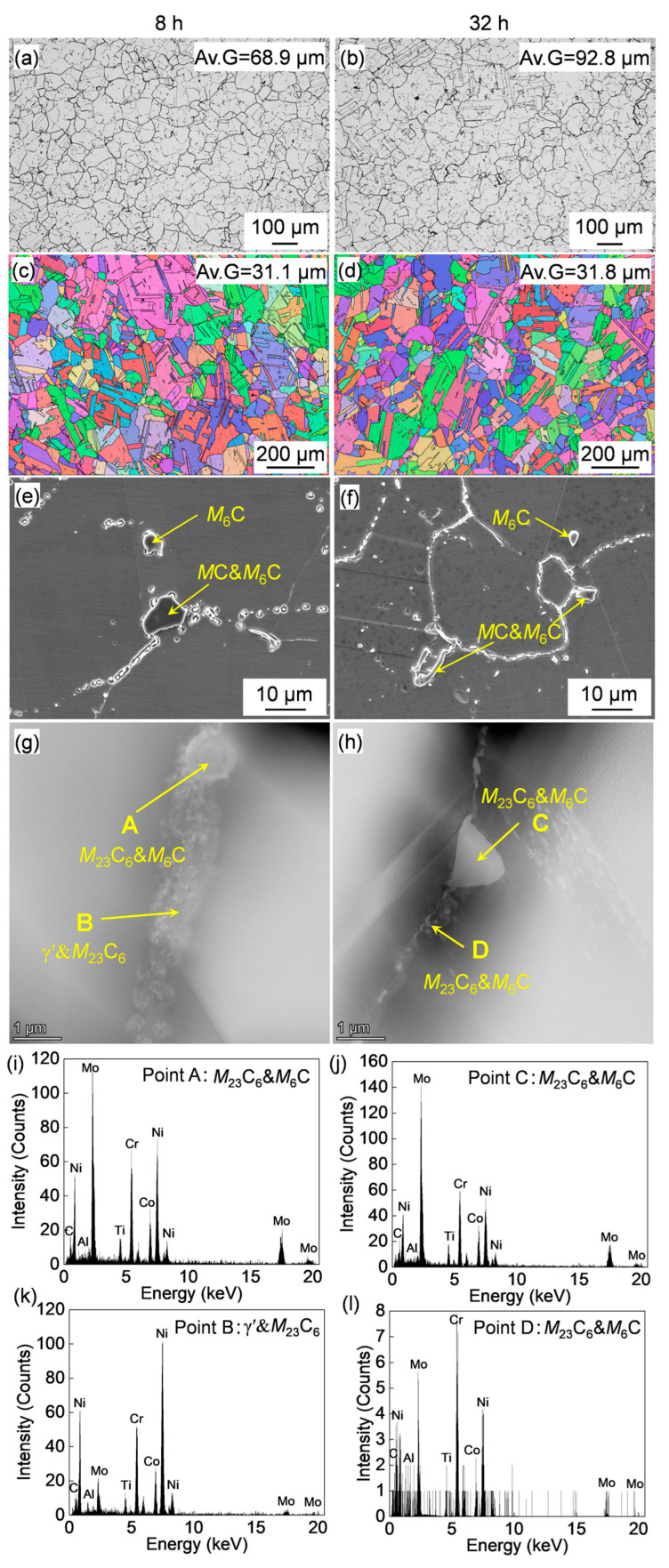
(**a**,**b**) OM, IPF maps (**c**,**d**) and (**e**,**f**) SEM images of the forged Rene 41 alloy after aging treated at 760 °C for varying periods. (**g**,**h**) HAADF images of the precipitates and (**i**–**l**) corresponding EDS point analyses results.

The OM images of the sample after aged at 760 °C for varying periods are shown in Figure 7a,b, the average grain size without consideration of twins is displayed as well. The average grain size of the alloy after aging for 8 h is similar with the that of 16 h (Figure 5b). While with the further increase in aging time to 32 h, the grain size was coarsened to 92.8 μm. Therefore, the 32-h aging period is relatively long and falls into the over-aging category in terms of grain size. In addition, a large number of annealing twins were observed as well after aging. The typical IPF image analyzed by EBSD and the average grain size with the consideration of twin boundaries were quantified using equivalent circular diameter method are shown in Figure 7c,d. The average grain size was significantly refined by the formation of twins. In addition, a large number of carbides were observed, which were analyzed by SEM (Figure 7c,d) and TEM analyses (Figure 7e–j). Fine carbides are distributed on the grain boundaries. Both the SEM and TEM observations confirmed that the carbides do not change significantly against aging time.

**Figure 8:**
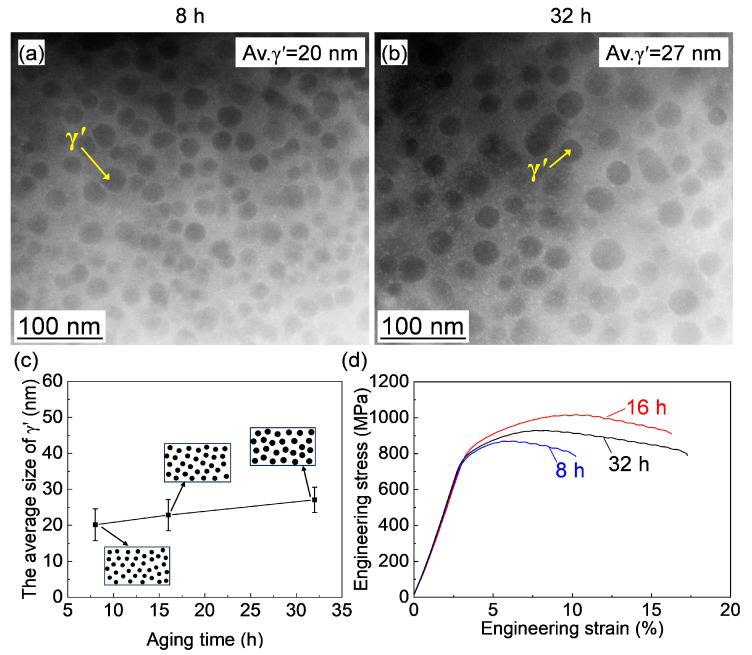
(**a**,**b**) HAADF images of the alloy aged at 760 °C for different durations; (**c**) Schematic illustrations showing the changes in γ′ morphology and size, as well as the quantified variation in γ′ size with aging time (the black dots represent γ′). (**d**) Stress–strain curves of the alloy aged at 760 °C for various periods.

The microstructure of γ′ after aging for varying periods was characterized by TEM, and typical HAADF images are shown in Figure 8a,b. Spherical γ′ particles were observed in all cases. The changes in size and morphology of γ′ are presented in Figure 8c. The γ′ size increased slightly with prolonged aging time at 760 °C, while the morphology remained almost unchanged. Compared to the effect of aging temperature, the aging time had a relatively weak influence on γ′. The resulting mechanical properties are shown in Figure 8d and Table 2. The yield strength initially increased and then decreased as the aging time extended from 8 h to 32 h. In contrast, the elongation continued to increase with aging time.

### 3.4. Strengthening Mechanisms

According to the above results, the microstructure and elevated-temperature mechanical properties of Rene 41 alloy are strongly influenced by the aging conditions. The yield strength and elongation can be appropriately alternated by aging. To understand the mechanical properties of Rene 41 alloy under varying aging conditions, the strengthening mechanisms needs to be well understood. A reliable strengthening model needs to be established to help quantitatively understand the strengthening mechanisms and the contributions to the overall yield strength. As for the wrought superalloy, the overall yield strength, *σ_y_*, are contributed by the intrinsic strengthening (*σ*_0_), grain boundary strengthening (*σ_Gb_*), solid solution strengthening (*σ_Ss_*), dislocation strengthening (*σ_Dis_*), and precipitation strengthening (*σ_Pre_*), which is expressed as [16,29]:

(1) $$\sigma_{y}=\sigma_{0}+\sigma_{Gb}+\sigma_{Ss}+\sigma_{Pre}+\sigma_{Dis}$$

The contribution of each is discussed and evaluated as follows.

(1) Intrinsic strength

The intrinsic strength for the pure Ni alloy can be calculated by the following equation [16,30]:

(2) $$\sigma_{0}=M\times \tau_{crss}$$

where *M* is the Taylor factor, which is taken as 3.06 for FCC [31]. $\tau_{crss}$ is the critical resolved shear stress. For nickel-based superalloy, $\tau_{crss}$ is 17.5 MPa [30]. The evaluated intrinsic strength is shown in Table 3, in which the experimentally measured yield strength for the alloy after aged at varying conditions is listed as well.

(2) Grain boundary strengthening

The grain boundary strengthening is mainly attributed to the impeding of the dislocation motion by the presence of high-density grain boundaries. The grain boundary strengthening of the nickel-based superalloy follows the Hall–Petch relationship, and it can be written as [32,33]:

(3) $$\sigma_{Gb}=k_{HP}\times d^{-1/2}$$

where *k_HP_* is the Hall–Petch constant, which is taken 710 MPa/μm^1/2^ for nickel-based superalloys [34], and *d* (μm) is the average grain size. As mentioned above, a large number of annealing twins were formed as well after aging. Twin boundary plays comparable role with that of grain refinement to increase the strength of alloys [19]. Therefore, the average grain size with the consideration of twin boundaries was used for the discussion of grain-boundary strengthening. The calculated grain-boundary strengthening (*σ_Gb_*) for the varying aging conditions is summarized in Table 3. The contribution of grain boundary strengthening (*σ_Gb_*) to yield strength is in the range of 125–170 MPa depending on the aging conditions and grain size. The grain-boundary strengthening was evaluated to be 147.6 MPa and 177.1 MPa for the vertical and horizontal directions, respectively, for the aged SLM IN 718 alloy [16], where the grain size (vertical: 23.15 μm, horizontal: 16.07 μm) is close to the present work. The grain-boundary strengthening was evaluated to be 130.5 MPa and 127 MPa, for the other nickel-based superalloys with an average grain size of 33 μm and 31 μm, respectively [35,36]. Therefore, the evaluated grain-boundary strengthening within 200 MPa for the current work with the grain size in the range of 20 μm~30 μm is reasonable.

(3) Solid solution strengthening

According to Labusch’s theoretical research [37], solute atoms in alloys can hinder the dislocation motion, thereby increasing the yield strength. The efficacy of solid solution strengthening originates from localized crystallographic distortions and elastic modulus disparities between the host lattice and incorporated solute species. Subsequently, Gypen et al. [38] extended this theory to multi-element alloys and established a solution strengthening model that accounts for the contribution of each element. The solid solution strengthening can be expressed as:

(4) $$\sigma_{Ss}=\left(1-f\right){\left[{\sum_{i}\left(\beta_{i}x_{i}^{1/2}\right)}^{2}\right]}^{1/2}$$

where f is the volume fraction of γ′; therefore, the volume fraction of γ matrix is 1 − f. The $x_{i}$ (at. %) represents the content of element *i* in the γ matrix, and $\beta_{i}$ (MPa/at. %) is the strengthening factor of solute element *i*, which is related to the atomic radius and modulus of element. The $\beta_{i}$ factor for the varying alloying elements was referenced from the literature [39], as shown in Table 4. In this study, it is assumed that the Al, Ti, and C elements have been fully consumed by the formation of γ′ and carbides after aging, and the B was ignored due to its trace content. Therefore, Cr, Co, and Mo were mainly considered for the solid–solution strengthening. The calculated solid–solution strengthening (*σ_Ss_*) is summarized in Table 3. The contribution of solid–solution strengthening (*σ_Ss_*) is in the range of 190–210 MPa, depending on the aging conditions. The solid–solution strengthening was evaluated to be 260.9 MPa for the aged SLM IN 718 alloy [16] with slightly higher solute content than the present work. In addition, the solution strengthening was approximately 200 MPa and 210 MPa for the hot extrusion FGH4113A [17] and FGH4097 [40] superalloys, respectively, with a close solute content with the present work. Hence, the solution strengthening evaluated in the present work is comparable with the previous works with close solute contents and reasonable.

(4) Dislocation strengthening

The interaction of mobile dislocations on the slip plane reduces their mobility, which leads to dislocation strengthening. The relationship between dislocation strengthening and dislocation density can be written as [41,42]:

(5) $$\sigma_{Dis}=M\alpha Gb\sqrt{\rho }$$

where *M* is the Taylor factor, which is taken as 3.06 for FCC [31], *α* is the empirical constant of nickel-based superalloy, equaling 0.2 [43]; b and G are the Burgers vector and shear modulus of γ matrix, respectively, taking as 0.248 nm and 80 GPa for nickel-based superalloy, respectively [31]. *ρ* is the dislocation density, which is generally 1 × 10^13^/m^2^ for the aged nickel-based superalloys [44,45]. The calculated dislocation strengthening (*σ_Dis_*) is also shown in Table 3. The evaluated dislocation strengthening (*σ_Dis_*) is 38.4 MPa, which is relatively small; therefore, it was ignored in some works for the aged superalloys [17,46]. Hence, the evaluated dislocation strengthening within 50 MPa is reasonable.

(5) Precipitation strengthening

Precipitation strengthening in wrought superalloys is the dominant strengthening mechanism that contributes to the overall strength. The principle of precipitation strengthening lies in impeding dislocation motion through nano-sized precipitates. It is widely accepted that precipitation strengthening depends on precipitate size. When dislocations interact with precipitates, small-sized precipitates primarily impede dislocations via the shearing mechanism (via the cutting mechanism), while large-sized precipitates form dislocation loops through the Orowan bypass mechanism, thereby increasing the resistance to dislocation motion [47,48,49]. The precipitation strengthening mechanism(s) in the variously aged alloys in this study are discussed by comparing with the experimentally measured yield strength and the above evaluated strengthening contributions.

The above calculated intrinsic strengthening (*σ*_0_), grain boundary strengthening (*σ_Gb_*), solid solution strengthening (*σ_Ss_*), and dislocation strengthening (*σ_Dis_*) are of the same order as those reported in previous studies with similar grain sizes, solute contents, and aged states. Therefore, the above strengthening evaluations are reliable, and a summary of these strengthening contributions is listed in Table 3. However, there remains a 300–400 MPa difference compared to the experimentally measured yield strength, as shown in Table 3. This difference is attributed to precipitation strengthening (*σ_Pre_*), which accounts for approximately 45% of the total contributions. γ′ precipitation strengthening is recognized as the primary strengthening mechanism for wrought nickel-based superalloys, contributing about 50% of the total strength at approximately 30 vol.% γ′ [17,34,50], which is consistent with the present findings.

**Figure 9:**
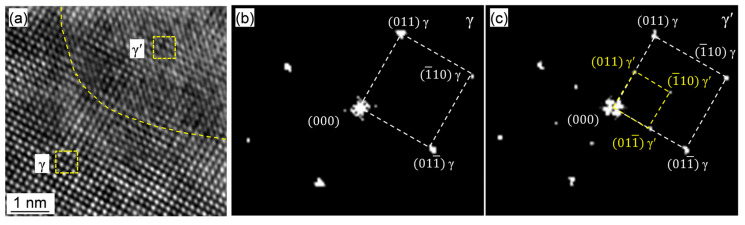
(**a**) High-resolution TEM image of Rene 41 after aging at 860 °C for 16 h and (**b**,**c**) the FFT analyzing results of the square regions in (**a**).

**Figure 10:**
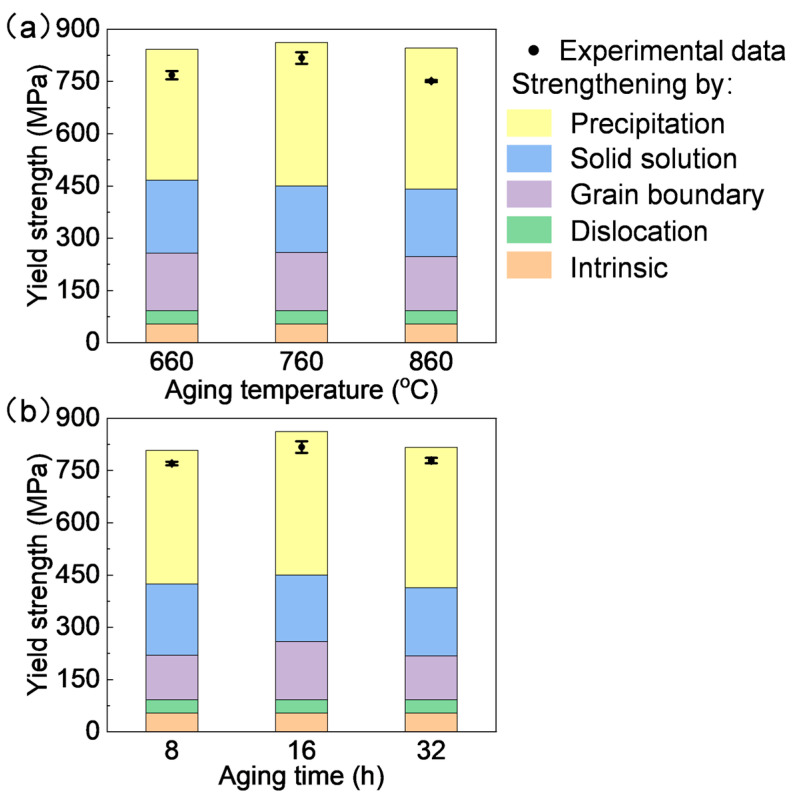
Comparisons of the predicted and experimentally measured yield strength after aged at (**a**) varying temperature and (**b**) aging period at 760 °C for Rene 41 alloy. Contributions from the five strengthening mechanisms are indicated by different colors.

The γ′ volume fraction for the Rene 41 alloy after aged at varying conditions were quantitatively measured by the point counting method based on the TEM analyses, and the corresponding results are shown in Table 5. As the aging temperature and aging time increase, the volume fraction of γ′ first increases and then slightly decreases, while the size of γ′ continuously increases (Figure 6 and Figure 8). The reduction in γ’ content at an elevated aging temperatures is attributed to the decrease in equilibrium fraction of γ’ against temperature according to the phase diagram shown in Figure 1a. It is known that the precipitation strengthening mechanism is strongly influenced by the coherency of the interface between γ′ and matrix [16]. It was reported that the square or spherical LI_2_-γ′ nanoparticles precipitate coherently on the FCC-γ matrix. The interface structure of γ’ and matrix was analyzed by TEM, by selecting a coarser γ′ formed after aging at 860 °C for 16 h. The typical high-resolution TEM (HRTEM) image is shown in Figure 9a. The diffraction spots of the matrix γ and γ′ obtained by the fast Fourier transform (FFT) are shown in Figure 10b,c. The γ’ holds a Cube–Cube O.R. ((100)_γ_∥(100)_γ’_, [010]_γ_∥[010]_γ’_) with the γ matrix. The HRTEM image clearly indicates that the γ/γ′ interface is coherent. This coherent interface remains stable even after high temperature aging. The lattice mismatch, δ, was calculated to be 0.019 based on the TEM analyses, which is relatively small, and thereby could be increasing the resistance to dislocation movement and enhancing the yield strength [51].

According to the precipitation strengthening theory, the critical resolved shear stress (CRSS), which governs the driving force necessary for dislocation pairs to cut through precipitates, directly determines the extent of precipitation strengthening. Based on the relationship between precipitate size and dislocation pair spacing, precipitation cutting mechanisms can be categorized into weak pair-coupling and strong pair-coupling [52]. Under shear stress, dislocations move as pairs along the slip plane from the γ matrix into γ′ precipitates. When the leading dislocation passes through a precipitate, an antiphase boundary (APB) is created; this APB is eliminated when the trailing dislocation passes through the precipitate [31,53]. For precipitates smaller than the dislocation pair spacing, the CRSS corresponds to the weak pair-coupling regime. When the precipitate size becomes comparable to the dislocation pair spacing, both dislocations may enter the precipitate simultaneously, corresponding to the strong pair-coupling case. The yield strength increments due to weak and strong pair-coupling are given by Equations (6) and (7), respectively [52,54].

(6) $$\sigma_{weak}=M\times \tau_{weak}=M\times \frac{\gamma_{APB}}{2b}\times \left(\sqrt{\frac{3\pi^{2}\gamma_{APB}fr}{32T}}-f\right)$$

(7) $$\sigma_{strong}=M\times \tau_{strong}=M\times 0.81\times \frac{\gamma_{APB}}{2b}\times {\left(\frac{3\pi f}{8}\right)}^{\frac{1}{2}}$$

where $\gamma_{APB}$ is the APB energy, which represents the threshold required for dislocation to shear through the precipitate, which is generally 0.126 J/m^2^ for nickel-based superalloys [55]. *r* represents the average radius size of γ′. *T* is the dislocation line tension, which is numerically equal to Gb^2^/2. Then, the critical radius *r_m_,* between the weak and strong cutting was calculated to be approximately 44.5 nm (89 nm in diameter) at the condition of $\sigma_{weak}=\sigma_{strong}$.

When the γ′ precipitate radius exceeds the critical value (*r_c_*), dislocations can no longer cut through the γ′ phase directly. Instead, they bend around the precipitate, forming Orowan loops to bypass it. The critical resolved shear stress (CRSS) for this Orowan mechanism (*σ_orowan_*) is expressed as follows [56]:

(8) $$\sigma_{orowan}=M\times \tau_{orowan}=M\times 0.8\times \frac{Gb}{L}$$

where $L={\left(\frac{2\pi }{3f}\right)}^{1/2}\times r$ [57], which is the average spacing of precipitates. Under the condition of *σ_orowan_*=*σ_strong_*, the critical radius *r_c_* was determined to be 138 nm (276 nm in diameter).

In this study, the average radius of γ′ under the aging condition of 860 °C for 16 h is larger than *r_m_* (Figure 6d), while the others are smaller than *r_m_* (Figure 6d and Figure 8c). This suggests that the precipitation strengthening mechanism may vary under different aging conditions. Therefore, the precipitation strengthening for the aged conditions with γ′ smaller than *r_m_* should be explained by the weak pair coupling (*σ_weak_*) theory.

The calculated precipitation strengthening values are summarized in Table 6 and compared with the expected precipitation strengthening (*σ_P__re_*) based on experimental measurements and the above discussions. Evidently, a significant discrepancy exists between the strength calculated using the weak pair-coupling theory (*σ_weak_*) and the expected value. In contrast, the yield strength can be well explained by the strong pair-coupling theory (*σ_strong_*). Similarly, the radius of γ′ precipitates after aging at 860 °C for 16 h falls between *r_m_* and *r_c_*, which is consistent with the strong pair-coupling theory (*σ_strong_*). The calculations demonstrate that the strengthening behavior is well described by the strong pair-coupling theory (*σ_strong_*), while notable discrepancies exist when using either the weak pair-coupling (*σ_weak_*) or Orowan bypass theories.

The comparison between the evaluated overall yield strength and experimental measurements for various aging conditions is shown in Figure 10. The predicted yield strength shows excellent agreement with experimental results, with an average relative error of 6.9% between predictions and experiments, demonstrating the reliability of the strengthening model. This consistency confirms the strengthening mechanisms in the Rene 41 alloy. For each aging condition, precipitation strengthening contributes approximately 50% to the overall yield strength (as indicated by the blue segments in Figure 10), representing the dominant strengthening mechanism in Rene 41 alloy, followed by solid solution strengthening and grain boundary strengthening.

It was reported that grain-boundary carbides retard grain boundary slip during plastic deformation [58]. The coarsened channels between carbides facilitate the accumulation of dislocations in these regions, which increases the alloy’s susceptibility to plastic deformation and enhances overall plastic strain. Additionally, carbides exert a pinning effect on grain boundary migration [59], thereby inhibiting grain growth and indirectly strengthening grain boundaries through refinement. Furthermore, micron-scale carbides are reported to predominantly improve high-temperature creep resistance [60].

**Figure 11:**
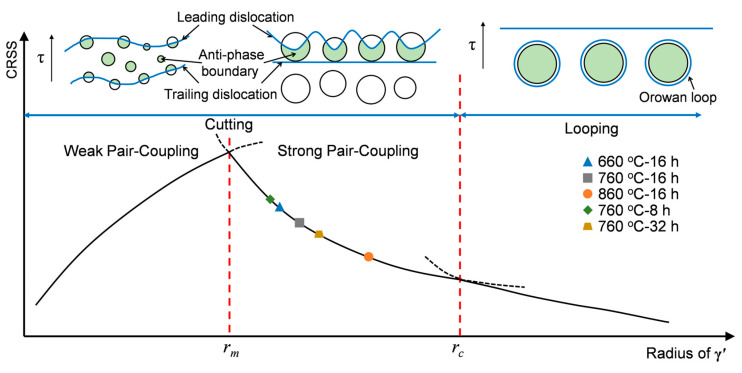
The strengthening mechanism of γ′ against its size.

The precipitation strengthening mechanisms of aged Rene 41 alloy are summarized in Figure 11. When the γ′ size is smaller than the critical size *r_c_*, dislocations primarily cut through γ′ precipitates. Conversely, when γ′ exceeds *r_c_*, dislocations mainly bypass the precipitates via the Orowan mechanism. Furthermore, when the γ′ radius surpasses another critical size *r_m_*, the dislocation motion is governed by the strong pair-coupling theory (*σ_strong_*); otherwise, the weak pair-coupling theory (*σ_weak_*) applies. In this study, with γ′ sizes ranging from 20 nm to 69 nm, all observed strengthening behaviors correspond to the strong pair-coupling regime (*σ_strong_*). Previous reports indicate that dislocations can more readily cut through γ′ precipitates with coherent interfaces, even for larger γ′ sizes [61,62,63].

The alloy aged at 760 °C for 8 h exhibits low yield strength (774.1 MPa) due to the incomplete γ′ precipitation. In contrast, aging for 32 h resulted in γ′ coarsening, leading to reduced strength and a slight increase in elongation (yield strength: 784.3 MPa; elongation: 17.2%). According to the cutting mechanism discussed above, γ′ coarsening facilitates a dislocation passage through the precipitates, thereby decreasing yield strength while enhancing ductility.

Under the current aging parameters (aging temperature ≤ 860 °C, time ≤ 32 h), the γ′ size is smaller than the critical size (*r_c_*) of 138 nm governing the transition from shearing to bypass mechanisms, which is relatively large due to the coherent interface between γ′ and the matrix [61,62,63]. The agreement between the theoretical calculations and experimental results confirmed that the dominant strengthening mechanism is shearing rather than Orowan by-passing. As suggested by Figure 11, γ’ coarsening, for example, obtained at elevated aging temperatures or prolonged aging times, would induce the transition of the strengthening mechanism from cutting to bypass.

This study systematically investigated the effects of aging on the microstructure and mechanical properties of Rene 41 alloy, providing critical guidance for manufacturing aerospace components such as turbine blades and combustion chambers. Experimental results demonstrate that aging at 760 °C for 16 h achieved an optimal balance between yield strength (823.5 MPa) and elongation (15.8%), offering direct references for industrial heat treatment parameter determination. Additionally, the research revealed that aging temperatures exceeding 760 °C or durations beyond 16 h induced grain coarsening and excessive γ′ phase growth, leading to strength reduction. These findings provide guidelines for determining optimal aging conditions to prevent service failures caused by over-aging. The experimental results and theoretical analyses offer essential support for the industrial fabrication of aerospace components using Rene 41 alloy.

## 4. Conclusions

1. As the aging temperature increased from 660 °C to 860 °C, the grain size gradually increased from 46.1 µm to 73.1 µm, and carbide distribution shifted from intragranular to grain boundaries. Additionally, the secondary γ′ phase size increased from 21 nm to 69 nm, with morphology evolving from spherical to quasi-square. The γ′ maintained a coherent interface with the matrix even after high-temperature aging at 860 °C. The yield strength initially increased and then decreased, while elongation continuously improved. Aging at 760 °C for 16 h achieved an optimal balance between strength and ductility.
2. At 760 °C, the grain size slightly increased when the aging time extended from 8 h to 16 h. However, significant grain coarsening occurred after 32 h, indicating excessive aging based on grain size evolution. The γ′ phase size marginally increased from 21 nm to 27 nm with a prolonged aging time, while maintaining a coherent interface with the matrix even after 860 °C aging for 16 h. The yield strength exhibited an initial rise followed by a decline, whereas elongation consistently improved.
3. Precipitation strengthening was identified as the dominant contributor to the overall yield strength (approximately 50%, 300–400 MPa), followed by solid solution strengthening (25%, 190–210 MPa) and grain boundary strengthening (20%, 125–170 MPa). Dislocation strengthening contributed minimally (<5%, ~38 MPa). The γ′ particle sizes ranged from 20 nm to 69 nm under the aging conditions of this study. Modeling confirmed that precipitation strengthening primarily arises from the strong pair-coupling cutting mechanism.

## Figures and Tables

**Table 1:** Chemical composition of Rene 41 superalloy [23] used in this work (mass%).

Cr	Co	Mo	Al	Ti	B	C	Ni
19.58	11.35	10.40	1.4	3.1	0.007	0.09	Bal.

**Table 2:** Mechanical properties of the alloys after aging under varying conditions.

	YS/MPa	TS/MPa	EL/%
660 °C–16 h	768.3 ± 12.1	884.6 ± 36.7	10.5 ± 1.3
760 °C–16 h	817.6 ± 16.5	975.2 ± 59.3	15.2 ± 1.6
860 °C–16 h	751.3 ± 2.8	802.3 ± 23.8	24.5 ± 0.4
760 °C–8 h	770.4 ± 5.3	860.7 ± 13.2	10.5 ± 0.4
760 °C–32 h	778.8 ± 7.8	907.3 ± 32.1	16.4 ± 1.2

**Table 3:** Comparisons of predicted strengthening contributions (*σ*_0_, *σ_Gb_*, *σ_Ss_*, *σ_Dis_*,) and experimentally measured yield strength (MPa).

	660 °C–16 h	760 °C–16 h	860 °C–16 h	760 °C–8 h	760 °C–32 h
Experiment YS	768.3 ± 12.1	817.6 ± 16.5	751.3 ± 2.8	770.4 ± 5.3	778.8 ± 7.8
*σ_0_*	53.6	53.6	53.6	53.6	53.6
*σ_Gb_*	165.5	167.4	155.1	127.3	126
*σ_Ss_*	209	190.7	194.6	205.4	195.8
*σ_Dis_*	38.4	38.4	38.4	38.4	38.4
*σ_0_ + σ_Gb_* + *σ_Ss_* + *σ_Dis_*	466.5	450.1	441.7	424.7	413.8
Expected *σ_p_*	301.8	367.5	309.6	345.7	365

**Table 4:** *β_i_* coefficients of different elements in γ matrix (MPa/at. %^1/2^) [39].

Cr	Co	Mo
337	39.4	1015

**Table 5:** Volume fraction of γ′ (*f*) after aging at varying conditions.

	660 °C–16 h	760 °C–16 h	860 °C–16 h	760 °C–8 h	760 °C–32 h
ƒ	0.303	0.364	0.351	0.315	0.347

**Table 6:** Theoretically calculated values of precipitation enhancement under different models (MPa).

	660 °C–16 h	760 °C–16 h	860 °C–16 h	760 °C–8 h	760 °C–32 h
Expected *σ_p_*	301.8	367.5	309.6	345.7	365
$\sigma_{weak}$	66.3	63.3	316	55.4	96.5
$\sigma_{strong}$	376.2	412.3	404.9	383.6	402.6
$\sigma_{orowan}$	1759	1760	576.3	1883	1464

## Data Availability

The raw data supporting the conclusions of this article will be made available by the authors on request.
